# Trees Are a Major Foraging Resource for Honeybees in the City

**DOI:** 10.3390/plants13213094

**Published:** 2024-11-03

**Authors:** Karen Koelzer, Alexandra Ribarits, Karin Weyermair, Johannes M. Bouchal, Josef Mayr, Martina Weber

**Affiliations:** 1 Division of Structural and Functional Botany, Department of Botany and Biodiversity Research, University of Vienna, Rennweg 14, 1030 Vienna, Austria; 2 Department for Apiculture and Bee Protection, Institute for Seed and Propagating Material, Phytosanitary Service and Apiculture, Division for Food Security, Austrian Agency for Health and Food Safety Ltd., Spargelfeldstraße 191, 1220 Vienna, Austria; 3 Department of Oto-Rhino-Laryngology, Medical University of Vienna, Währinger Gürtel 18–20, 1090 Vienna, Austria; 4 Department for Statistics and Analytical Epidemology, Division for Data, Statistics and Risk Assessment, Austrian Agency for Health and Food Safety Ltd., Zinzendorfgasse 27/1, 8010 Graz, Austria

**Keywords:** floral resources, honey, melissopalynology, plant diversity, pollen analysis, pollinators, urban ecology

## Abstract

Large cities are typically characterized by a mosaic of green spaces that hold a remarkable variety of native and “exotic” plants. Urban beekeeping has gained increasing popularity. In order to characterize the “urban” in the honey, pollen diversity in 50 honey samples from 18 apiary locations in Vienna, Austria, was microscopically analyzed. The relative abundances of each plant taxon were determined by counting out 500 individual pollen grains per sample. In total, 202 taxa could be identified, with a median of 46 per sample. Taxa richness and diversity differed significantly across three years but did not so between urban and suburban apiaries. Despite trees comprising only roughly a quarter of all taxa, the amount of tree pollen was disproportionally high. The invasive *Ailanthus altissima* was predominant in 15 out of 50 samples. Other important non-native and/or ornamental trees included *Sophora japonica*, *Gleditsia triacanthos*, *Castanea sativa*, *Koelreuteria paniculata*, and *Liriodendron tulipifera*. Urban honey from Central Europe may typically comprise pollen taxa from Europe, East Asia, and North America alike. The results of this study show that intentionally planted, managed urban green spaces can support stable foraging resources for pollinators in cities.

## 1. Introduction

Urban landscapes are characterized by the heterogeneity of floral resources and habitats [1,2]. However, the high proportion of sealed surfaces is an inhospitable matrix for many pollinators. In addition, exposure to higher temperatures in “urban heat islands” affects not only the physiology of plant species but also the quality of plant–pollinator interactions [3]. Worldwide, a decline in pollinators could be linked to human pressures, environmental change, and a high degree of fragmentation [4,5].

Plants in the city are vital for humans and animals in many aspects. In view of global warming, trees, in particular, have become an important source of shade for residents, besides fulfilling an aesthetic purpose. At the same time, they act as a valuable food source for pollinating insects. Appropriately managed urban green spaces can act as hotspots for pollination services, specifically for bees [6,7].

Honeybees of the *Apis mellifera* subsp. *carnica* race, which is the most frequently kept honeybee type in Austria [8], are generalist flower visitors. They collect pollen and store it inside the hive as beebread (perga), which provides the larvae with protein. Nectar is stored in the comb cells as honey and is the colony’s primary source of sugar. Pollen grains are indirectly introduced to honey through secondary input in the beehive [9,10]. The specific morphology and characteristics of pollen grains allow us to microscopically identify their taxonomic source and hence link the honey to the botanical and geographical source of honeybee foraging [11,12].

Urban beekeeping has many benefits: It contributes to a healthy environment by maintaining plant–pollinator interaction in urban areas, provides economic profits for beekeepers and apiary localities, and is a valuable tool to educate the general public about the importance of pollinators [13,14]. There is a continuous trend to keep honeybees in urban environments. Yet, the authenticity of urban honey from a melissopalynological perspective is often unclear.

Urban landscapes present particular challenges for melissopalynological pollen analyses due to a high diversity of both native and ornamental plants [15]. Ornamentals, especially, have become a vital part of municipal and private planting regimes [16]. Typically, certain “marker” pollen types indicate the origin of a honey sample by knowing the geographical distribution of that plant taxon. As relatively few plant individuals can provide vast amounts of flowers, trees especially comprise an important food source for bees in disturbed areas such as urban environments [17,18]. In recent years, however, tree species from other geographical regions have been widely introduced to Central European cities as inner-city environments increasingly mimic the heat and drought conditions of their original distributions. Those trees include, e.g., *Ailanthus altissima*, *Koelreuteria paniculata*, and *Sophora japonica* from the Qinling Mountains in China, *Gleditsia triacanthos* and *Liriodendron tulipifera* from the Appalachians in the United States, and *Tilia tomentosa* from the East European steppes [19,20]. When available in higher abundances, introduced and non-native plants may also change the seasonal pattern of foraging resources for pollinators [3].

A more systematic approach is essential to shed knowledge on the spatial and temporal availability of floral resources in cities and to understand honeybee preferences. The aim of this study is to gain basic knowledge of the actual foraging resources in a Central European urban metropolis and highlight the importance of planted trees for honeybees. It examines the availability and origin of trees across the heterogeneous mosaic of urban and suburban landscapes. Knowing how honeybees interact with the floral resources in their environment will provide information for the management of municipal planting regimes, as well as point private owners of gardens towards honeybee-friendly plant choices. Moreover, this study will give scientific guidance for beekeepers regarding the spatial and temporal availability of floral resources in a city and how they might impact hive management.

## 2. Results

**Figure 1:**
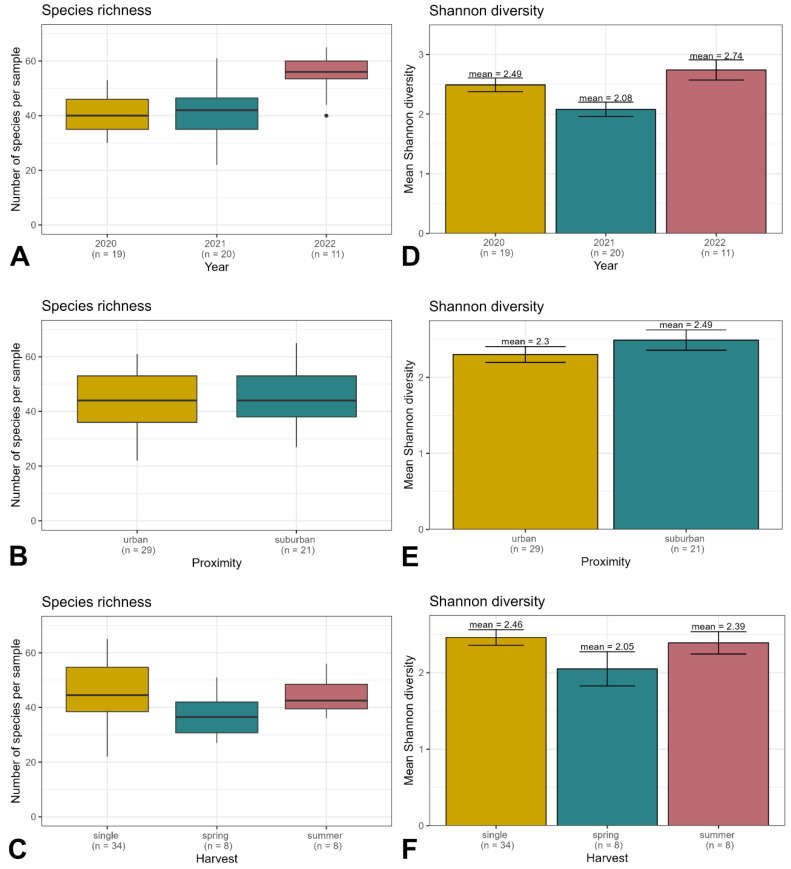
(**A**–**C**): Taxa richness per sample across three years (2020–2022; (**A**), by proximity (urban and suburban; (**B**), and three harvesting times (single harvest in June, spring harvest in the first half of July, and summer harvest in July–August; (**C**). (**D**–**F**) Mean Shannon Diversity Index of pollen spectra across years (**D**), proximity (**E**), and harvesting times (**F**).

For this study, 50 honey samples were melissopalynologically analyzed. This includes nineteen honey samples in 2020, twenty samples in 2021, and eleven samples in 2022. Across all samples and all three years, a total of 202 individual pollen types could be identified. Out of these, 52 pollen types could be identified at the species level (25.7%), 91 at the genus level (45%), and 23 at the family level (11.4%). A further 5 could be identified down to two or three potential genera (2.5%), 25 were classified as “type” (12.4%), and 6 remained unidentified (3%). Across all years, the mean number of taxa per sample was 46. The mean number of taxa was highest in 2022 (Figure 1A). In general, the number of identified plant taxa did not correlate with the number of apiary locations per year (see Table 1).

Mean taxa richness and mean Shannon Diversity Index were calculated for the different years (Figure 1A,D), the urban and suburban groups (Figure 1B,E), and the different harvesting times (Figure 1C,F). The diversity of foraged plants was significantly different between the years (Table 1) but did not significantly change from urban to suburban locations and between extraction times. Differences in taxa composition were assessed by permutational multivariate analysis of variance, which revealed significant differences by year, urban vs. suburban location, and extraction time.

**Figure 2:**
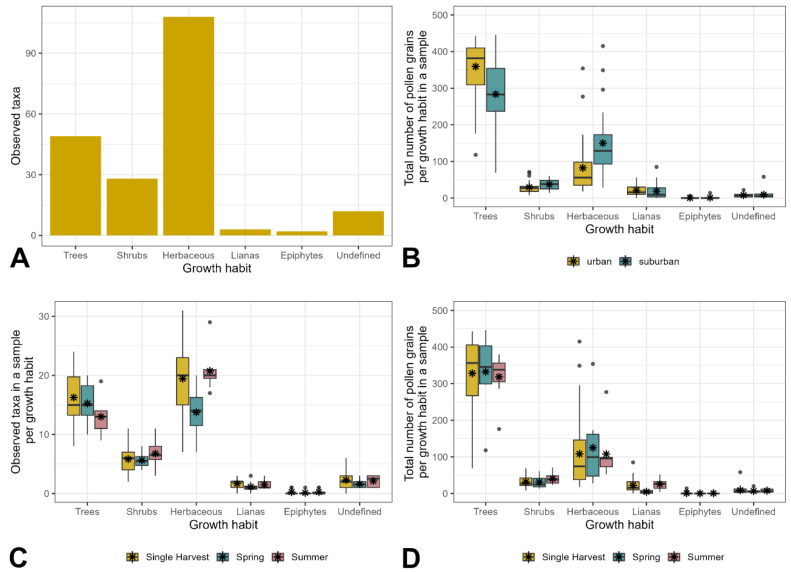
Number of identified taxa per growth habit (**A**), total amount of pollen per growth habit per 500 counted grains in the urban and suburban groups (**B**), distinct taxa counts per growth habit at different harvesting times (**C**), total amount of pollen grains per growth habit per 500 counted pollen grains at different harvesting times (**D**). Asterisks indicate the mean value across all 50 honey samples.

Further, a growth type was assigned to each of the 202 plant taxa, if possible. As shown in Figure 2A, they belong to herbaceous species (108), trees (49), shrubs (28), lianas (3), epiphytes (2), and various growth types (12). The number of tree taxa was clearly lower than the number of herbaceous species. Despite that, the relative abundance of tree pollen per 500 counted pollen grains by far exceeded all other categories (Figure 2B), with a median of 350. This trend was reproducible in 2020 and 2021 across all three harvesting times (single harvest, spring harvest, and summer harvest); see Figure 2C,D. 

**Figure 3:**
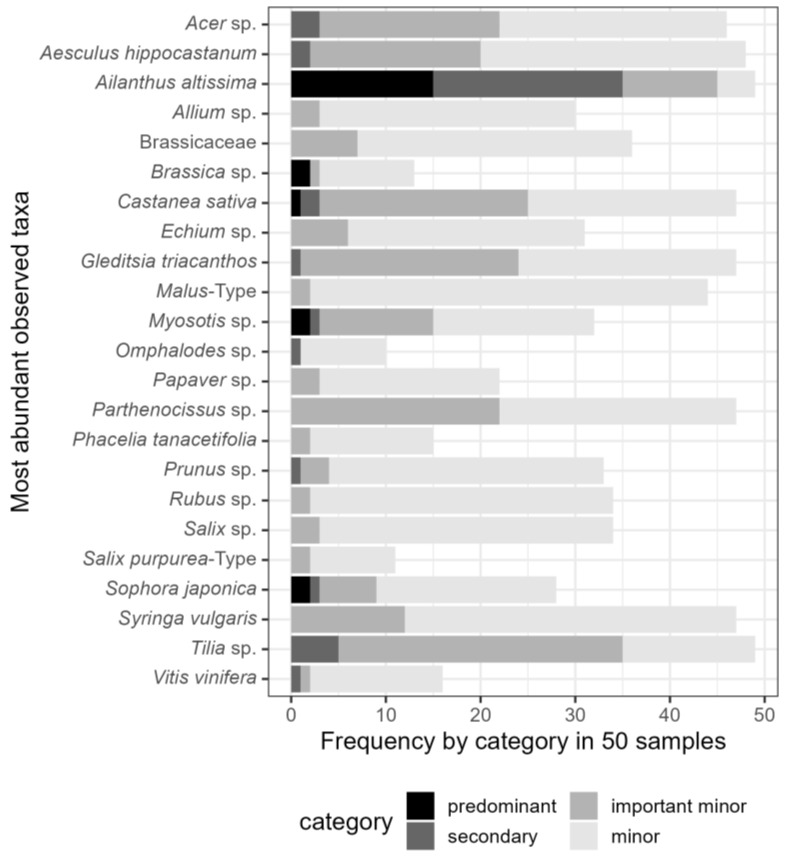
Pollen types with more than 8% abundance in at least one of the 50 honey samples. The occurrence is displayed according to defined frequency categories (predominant = > 45%, secondary = 15–45%, important minor = 3–15%, minor = < 3%).

**Figure 4:**
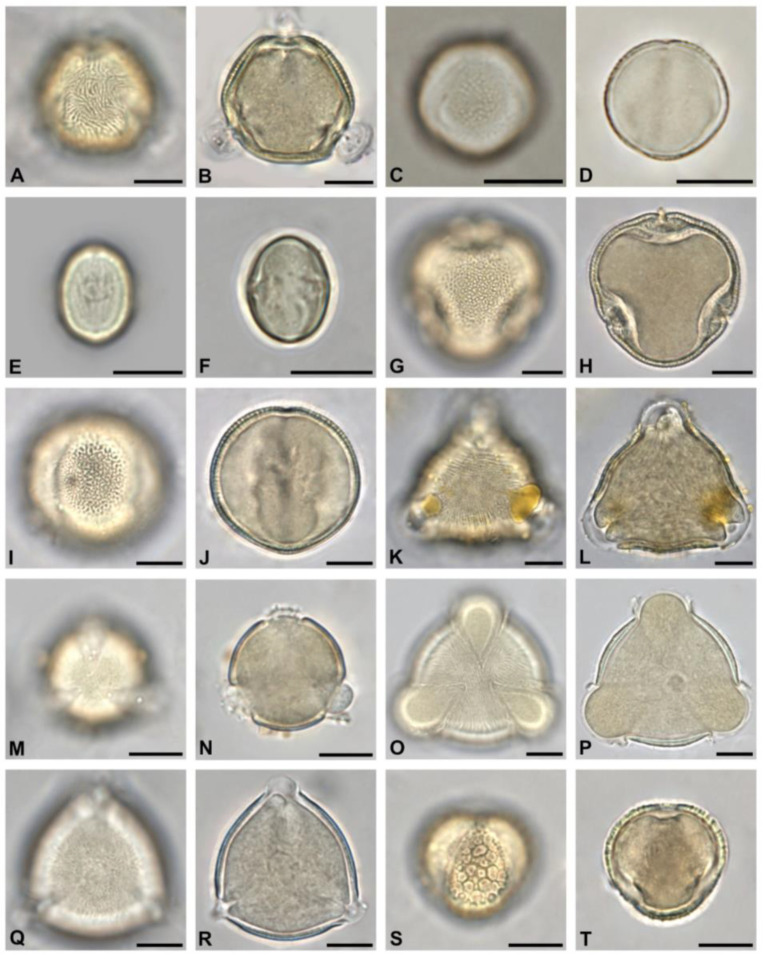
The thirty most abundant tree pollen types in honey from Vienna, Part I: *Ailanthus altissima* in polar view (**A**,**B**), *Sophora japonica* in equatorial view (**C**,**D**), *Castanea sativa* in equatorial view (**E**,**F**), *Tilia* sp. in polar view (**G**,**H**), *Gleditsia triacanthos* in equatorial view (**I**,**J**), *Prunus* sp. in polar view (**K**,**L**), *Aesculus hippocastanum* in polar view (**M**,**N**), *Acer* sp. in polar view (**O**,**P**), *Malus*-Type in polar view (**Q**,**R**), and *Salix* sp. in equatorial view (**S**,**T**). Scale bar indicates 10 µm.

**Figure 5:**
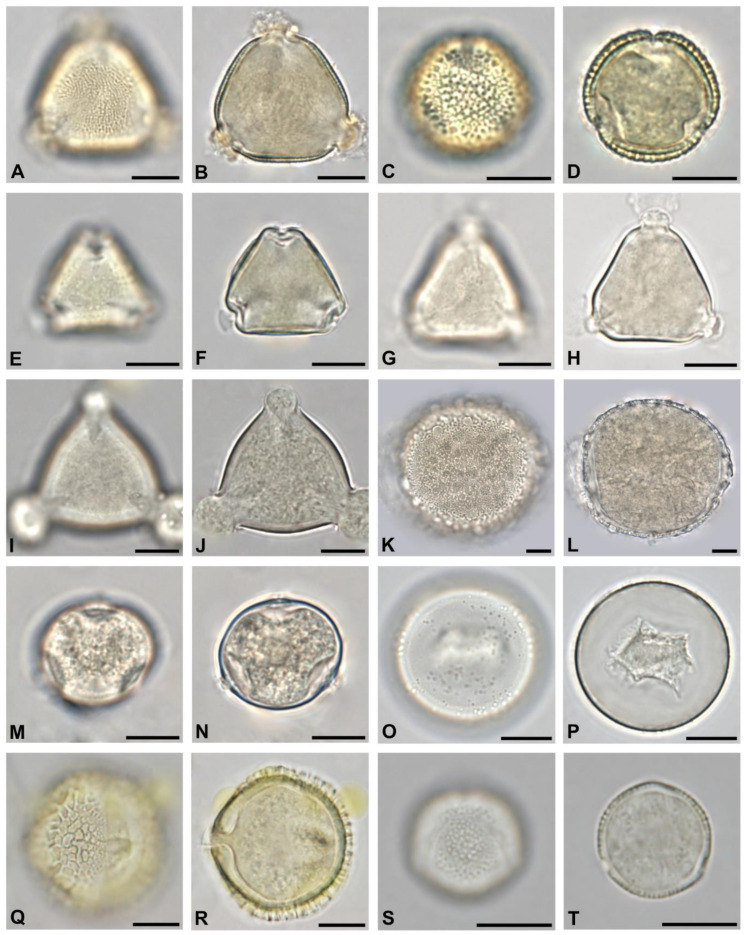
The thirty most abundant tree pollen types in honey from Vienna, Part II: *Koelreuteria paniculata* in polar view (**A**,**B**), *Fraxinus ornus* in polar view (**C**,**D**), *Rhamnus* sp. in polar view (**E**,**F**), *Frangula* sp. in polar view (**G**,**H**), *Robinia pseudoacacia* in polar view (**I**,**J**), *Liriodendron tulipifera* in polar view (**K**,**L**), *Morus* sp. in polar view (**M**,**N**), *Taxus* sp. (**O**,**P**), *Tetradium danielii* in equatorial view (**Q**,**R**), and *Tamarix* sp. in equatorial view (**S**,**T**). Scale bar indicates 10 µm.

**Figure 6:**
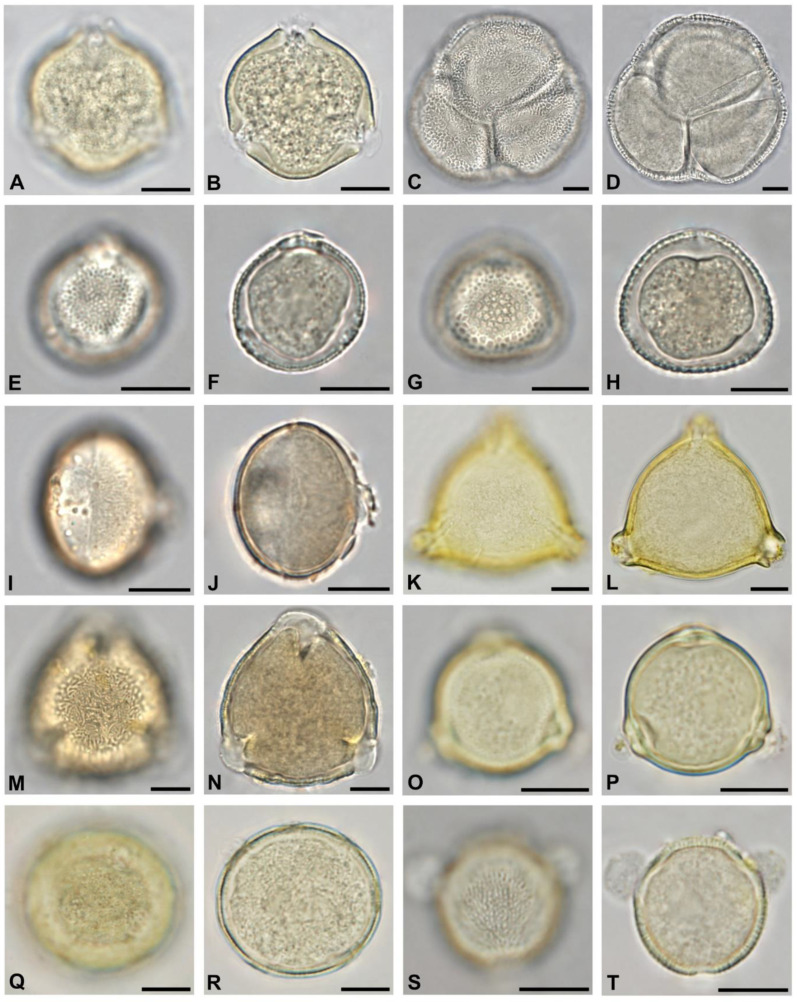
The thirty most abundant tree pollen types in honey from Vienna, Part III: *Quercus* sp. in polar view (**A**,**B**), tetrad of *Catalpa* sp. (**C**,**D**), *Platanus* sp. in polar view (**E**,**F**), *Fraxinus excelsior* in polar view (**G**,**H**), *Aesculus* x *carnea* in equatorial view (**I**,**J**), *Elaeagnus angustifolia* in polar view (**K**,**L**), *Prunus domestica* in polar view (**M**,**N**), *Betula* sp. in polar view (**O**,**P**), *Liquidambar styraciflua* (**Q**,**R**), and *Cotinus coggygria* in polar view (**S**,**T**). Scale bar indicates 10 µm.

**Figure 7:**
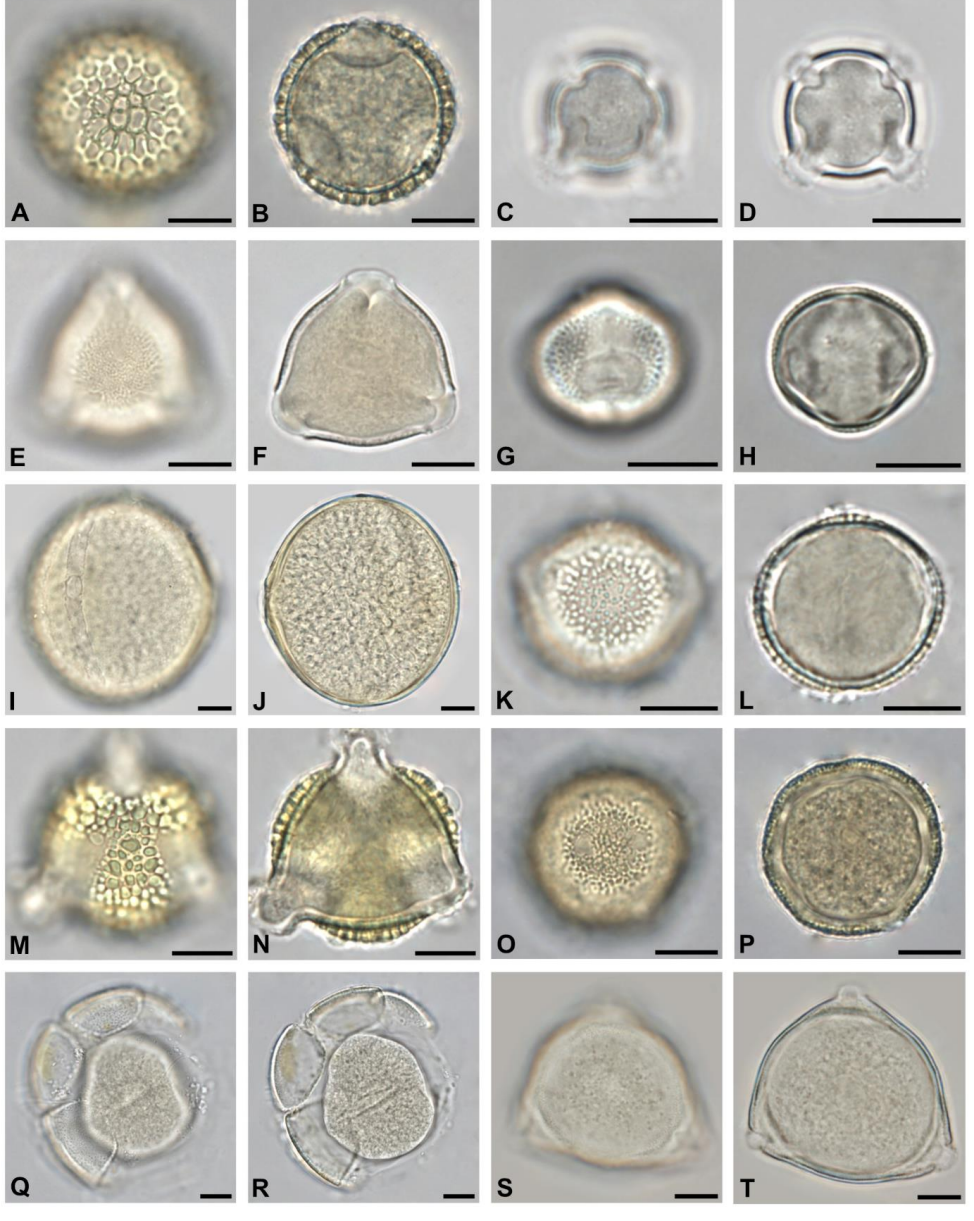
The ten most abundant pollen types of shrubs in honey from Vienna: *Syringa* sp. in polar view (**A**,**B**), *Buddleja* sp. in polar view (**C**,**D**), *Rubus* sp. in polar view (**E**,**F**), *Sambucus*-Type in equatorial view (**G**,**H**), *Cornus sanguinea* in equatorial view (**I**,**J**), *Viburnum lantana*-Type in equatorial view (**K**,**L**), *Ilex aquifolium* in polar view (**M**,**N**), *Buxus sempervirens* (**O**,**P**), *Berberis* sp. (**Q**,**R**), and *Symphoricarpos* sp. in polar view (**S**,**T**). Scale bar indicates 10 µm.

To determine the overall most abundant taxa, all pollen types with more than 8% relative abundance in at least one sample were considered (Figure 3). Out of these 23 taxa, ten belonged to trees. They were *Acer* sp., *Aesculus hippocastanum, Ailanthus altissima*, *Castanea sativa*, *Gleditsia triacanthos*, *Malus*-Type, *Prunus* sp., *Salix* sp., *Sophora japonica*, and *Tilia* species. Two were shrubs (*Rubus* sp. and *Syringa* sp.), and two were lianas (*Parthenocissus* sp. and *Vitis vinifera*). The rest was herbaceous or of various growth types. Only five identified pollen types occurred predominantly (>45%) in at least one honey sample. These were *Ailanthus altissima*, *Castanea sativa*, *Myosotis* sp., *Sophora japonica*, and *Brassica* species. Micrographs of the most occurring tree taxa are shown in Figure 4, Figure 5 and Figure 6, and of the most occurring shrubs in Figure 7.

The seven taxa most widely occurring across all 50 honey samples (see Table 2) were *Ailanthus altissima*, *Tilia* sp., *Aesculus hippocastanum*, *Gleditsia triacanthos*, *Parthenocissus* sp., *Syringa* sp., and *Castanea sativa*. *Ailanthus altissima* pollen (Figure 4A,B) occurred in 49 out of 50 honey samples and at all 18 apiary locations. Its highest relative abundance per 500 counted pollen grains was 80.6%, with a mean of 31%. It was the predominant pollen type (>45%) in 15 out of 50 samples. Pollen of *Tilia* sp. was likewise present in 49 out of 50 honeys and at all 18 apiary locations but was predominant in neither sample. The relative abundance of *Tilia* sp. pollen grains was highest at 37.2%, with a mean of 6.6%.

To examine the importance of non-native and/or ornamental species, the identified taxa were assigned to one or more of the following four categories: established/native to Austria, invasive in Austria, ornamental species, and agricultural species. The majority of taxa were native/established (145), followed by ornamental (71), agricultural (13), and invasive species (12). *Ailanthus altissima*, for example, was included in the established and invasive categories.

Out of the 49 identified tree taxa, 19 were non-native ornamentals (see Table 3). Those occurring in more than half of all analyzed honey samples and locations were *Sophora japonica* (Figure 4C,D), *Gleditsia triacanthos* (Figure 4I,J), *Koelreuteria paniculata (*Figure 5A,B), and *Liriodendron tulipifera* (Figure 5K,L).

Pollen of nectarless species was included to cover the full diversity of botanical origin and occurred in all 50 honey samples. Neither the apiary location in urban or suburban areas nor the harvesting time did affect the abundance of nectarless taxa significantly. The most frequent pollen of nectarless trees was of *Betula* sp. (Figure 6O,P), *Morus* sp. (Figure 5M,N), *Pinus* sp., *Platanus* sp. (Figure 6E,F), *Quercus* sp. (Figure 6A,B), and *Taxus* sp. (Figure 5O,P). None of these taxa occurred with more than 3% relative abundance in any of the samples.

### 2.1. Urban and Suburban Sites

According to the proximity to the city center and the amount of dense grayscape within the 3 km flight radius, 29 honey samples were classified as from urban locations and 21 as from suburban locations (Table 4). In the “urban” group, 167 out of 202 pollen types were observed, while the “suburban” group contained 164 out of 202 pollen types. The mean number of taxa per sample was similar between the two groups (Figure 1B). Taxa richness between the urban and suburban groups differed non-significantly across the years, with the largest variation in 2021.

The amount of tree pollen with respect to the number of tree taxa was disproportionally high in urban as well as suburban sites (Figure 2A,B). The median number of pollen from herbaceous species per sample was considerably higher in suburban areas than in urban areas (Figure 2B).

The number of ornamental plant taxa, as well as the relative abundances of ornamentals’ pollen per 500 counted grains, was higher in urban sites closer to the city center; however, the differences were not statistically significant.

### 2.2. Time and Frequency of Honey Extraction

In the years 2020 and 2021, honey from four locations was extracted twice. The spring harvest (indicated by “1” in Figure 1 and Figure 2) took place in the first half of June. The summer harvest (indicated by “2”) followed at the end of July. All other honey samples were derived from a single harvest in the first half of July (indicated by “0”). General taxa richness was higher in later extraction times compared to honey harvested in spring (Figure 1C). The mean number of identified tree taxa per sample, however, decreased from 15 in the first harvest to 13 in the second harvest (Figure 2C). The least number of herbaceous taxa occurred in spring. At all harvesting times, the relative abundances of tree pollen were disproportionally higher compared to the number of tree taxa (Figure 2C,D). The frequency of tree pollen per 500 pollen grains was similarly high in honey from spring, summer, and a single harvest.

### 2.3. Comparison of Four Different Locations Across Three Years

**Figure 8:**
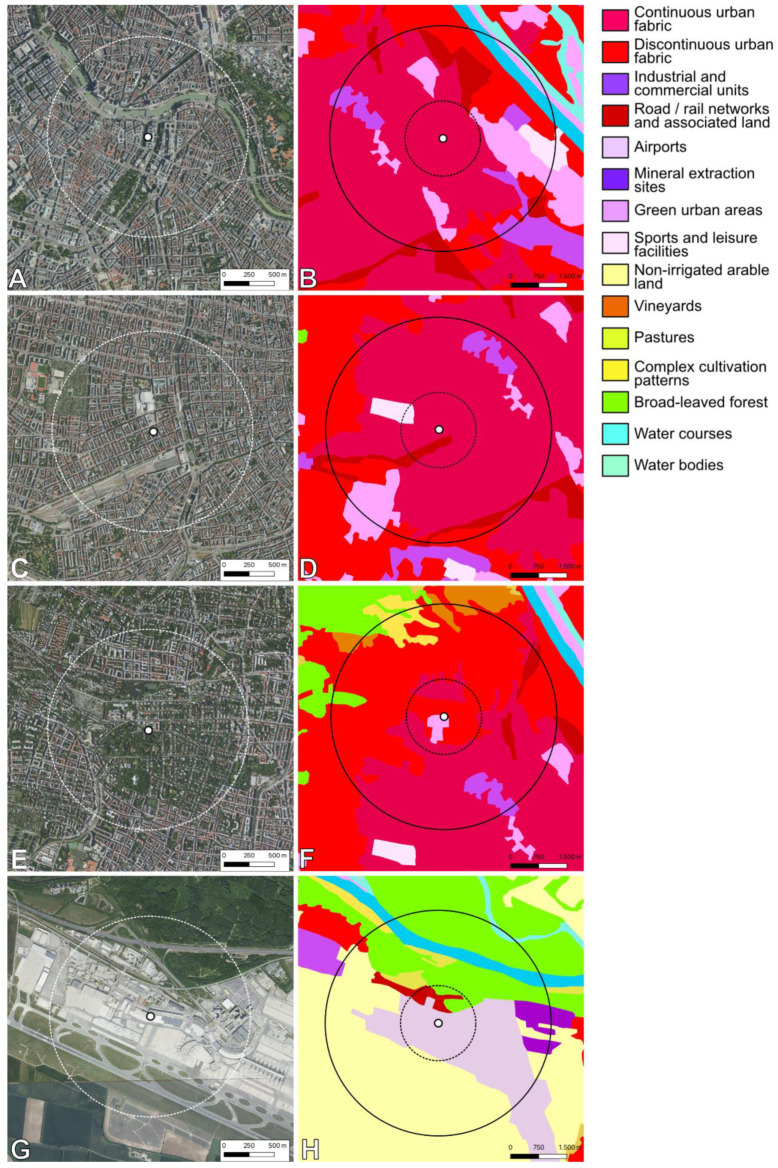
Selected apiary locations and the surrounding landscapes as ortho-photo with a 1 km radius (left), as well as the CORINE land cover analysis with a 1 km and 3 km radius (right): 1st district Stubenring (**A**,**B**), 15th district Boutiquehotel Stadthalle (**C**,**D**), 18th district University of Natural Resources and Life Sciences (**E**,**F**), and Vienna International Airport (**G**,**H**).

Four apiary locations were selected for a more detailed look at pollen diversities, as they represent the gradient from densely built-on urban areas to more open suburban landscapes. They were in the 1st and 15th districts of Vienna, as well as the 18th district and the airport (Figure 8).

**Figure 9:**
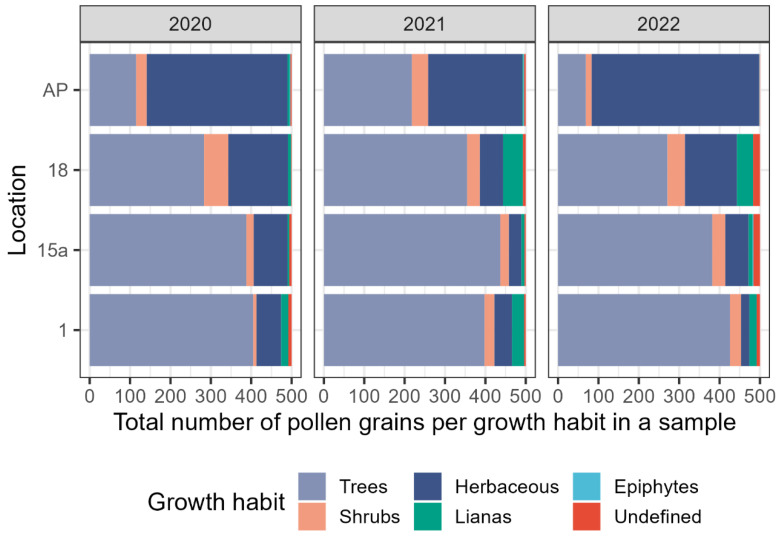
Total amount of pollen per growth habit per 500 counted grains at four selected locations: 1st district Stubenring (1), 15th district Boutiquehotel Stadthalle (15a), 18th district University of Natural Resources and Life Sciences (18), and Vienna International Airport (AP).

The amounts of tree pollen within 500 counted pollen grains were highest at the city center and decreased towards the outskirts. At all locations except for the airport, pollen of trees occurred in disproportionally high amounts (see Figure 9).

The mean number of identified pollen types across all three years was highest in the 18th district (51) and lowest in the 1st district (40). The highest number of tree taxa occurred in the 18th district in the year 2022 (24). The least number of tree taxa were identified in honey from the airport, with only eleven taxa in the year 2020. Similarly, the relative abundance of tree pollen in honey from the airport was low, with a minimum of 13.8% in the year 2022. This was the only sample out of 50 where the amount of counted tree pollen was negatively correlated to the number of tree taxa.

The most frequent tree pollen types in the 1st and 15th districts were *Ailanthus altissima* and *Tilia* species. They occurred, among others, together with *Acer* sp., *Aesculus hippocastanum*, *Gleditsia triacanthos,* and *Koelreuteria paniculata*. Towards the outskirts, the amounts of *Ailanthus altissima* and *Tilia* pollen decreased and the amount of *Castanea sativa*, *Malus*-Type, *Prunus* sp., and *Salix* pollen increased. The most frequent herbaceous species at the four selected locations were Brassicaceae, *Myosotis* sp., *Echium* sp., and *Phacelia tanacetifolia*. In addition, a high frequency of *Brassica* sp. pollen was found in honey from the airport and considerable amounts of *Allium* sp. in the 18th district.

## 3. Discussion

Over a period of three years, pollen diversity in 50 honey samples from 18 different locations in Vienna was analyzed microscopically in order to investigate the foraged plants. Tree taxa were shown to be disproportionally more abundant in honey from urban areas. Potentially, relatively few plant individuals provide mass amounts of nectar and pollen throughout the season.

### 3.1. The Role of Trees as Floral Resources

From the top twelve most planted tree species according to the Statistical Yearbook of the City of Vienna [21], seven were also among the most abundant taxa found in the honey samples (Figure 3). They were *Acer*, *Tilia*, *Aesculus* (identified as *A. hippocastanum* and *A.* x *carnea*), *Prunus*, *Pyrus* (identified as *Malus*-Type), *Gleditsia triacanthos*, and *Sophora japonica*. Some of those tree species were planted throughout the 20th century all across town, such as *Acer* sp. and *Tilia* species. *Gleditsia triacanthos* has also been growing along urban and suburban roads for some decades. *Aesculus hippocastanum* has been a popular urban tree for centuries because of its majestic size and inflorescences of ornamental value. *Aesculus* x *carnea* varieties and *Aesculus flava* are planted far less commonly but with increasing tendency [22].

*Fraxinus*, *Platanus*, and *Robinia pseudoacacia* (Figure 5I,J), which are also frequently planted according to tree records [21], occurred in lesser but still considerable amounts of honey. Other tree species have become popular in the past twenty years, and many individuals have yet to reach a maximum crown size. Those include e.g., *Sophora japonica*, *Liriodendron tulipifera*, and *Koelreuteria paniculata*. *Sophora* was shown to be the predominant pollen type in two samples. Thanks to its high nectar availability, *Sophora japonica* has the potential to be a major floral resource for honeybees wherever it is regularly planted. Pollen from *Liriodendron* and *Koelreuteria* occurred in more than half of all analyzed honey samples, also indicating potential use for the melissopalynological identification of urban origin. *Castanea sativa* is very rarely considered by municipal landscapers yet is sometimes planted by private owners of adequately large gardens.

The availability of nectar-producing trees might positively impact honeybees in particular, as they are polylectic generalists who prefer exploiting mass foraging resources, if available [9,23]. During the flowering season of common tree species in parks and alleys, they find large amounts of food in one place at the same time. Common tree species such as *Tilia* sp. and *Robinia pseudoacacia* are popular sources of monofloral honey in Austria. Their role as mass foraging plants might benefit apiarists as well. Further, the high diversity of trees in cities provides food throughout the season.

In pollen pellets, a decrease in the abundance of tree pollen was observed over the season [24]. In contrast, this study shows that in honey and especially urban honey, the amount may remain stable. The flowers of native trees like *Salix*, *Acer*, *Prunus, Rhamnus* (Figure 5E,F), and *Frangula* (Figure 5G,H) are succeeded by *Tilia* and *Ailanthus* in June–July, followed by *Koelreuteria* and *Sophora* in July–August.

### 3.2. Mass Foraging Plants

In contrast to most wild bees, honeybees prefer the exploitation of mass foraging sources, regardless of whether they are managed or not [25]. As the predominant species in this study underline, these can be agricultural crops or widely planted urban trees of native and non-native origin.

*Ailanthus altissima* is fast-propagating and tolerant against drought as well as industrial pollutants [26], which makes it well adapted to urban conditions. In this study and perhaps typical for urban apiaries, *Ailanthus altissima* occurred alongside *Tilia* sp., which is also a very common provider of monofloral honey. In the Statistical Yearbook of Vienna [21], *Ailanthus altissima* is mentioned with less than 500 individuals. However, as an invasive neophyte, *Ailanthus altissima* grows in many crevasses and corners in inner city areas, as well as alongside side roads and rail tracks. Those trees likely provide the majority of *Ailanthus* pollen in honey. They are not intentionally planted, yet they are an essential source of nectar and pollen for honeybees and other urban pollinators. This, as well as the mass foraging of “exotic” urban tree species such as *Sophora japonica,* shows the quick process of adaption in honeybees considering new mass foraging resources. The nectar value of *Ailanthus altissima* is 3 (out of 4), with more sugar produced in male flowers [26]. Additional extra-floral nectaries attract pollinators [27]. The main flowering time is June to July. So far, very limited literature on *Ailanthus altissima* as a potential source of monofloral honey is available [28]. Honey is said to be of amber color and crystallizes relatively early. The taste has been described as stingingly fruity, resembling the odor of Muscat [29].

*Tilia* pollen was most abundant in the year 2021, thanks to favorable weather conditions in July at the time of flowering. It peaked in the 1st and 18th districts. Honeybees foraged the numerous *Tilia* trees, which are planted along the Vienna Ring Road, as well as other parks and roadsides. Additionally, in the outskirts of the 18th district, *Tilia* trees occur naturally in the broadleaved forest of the Wienerwald. According to the online tree register of the city of Vienna [22], as many as six distinct species of *Tilia* and four hybrids are intentionally planted within the borders of Vienna.

Besides trees, the most abundant taxa were *Brassica* and *Myosotis*. Each of these was predominant in two honey samples. *Brassica* species are regularly cultivated in the agricultural areas on the north-eastern and south-eastern outskirts of Vienna and were also identified predominantly in honey samples from this area (11th district and airport). *Myosotis* is a common genus of the ground cover. Among the widely identified taxa across all samples were *Plantago* and the climber *Parthenocissus*, which were present in almost all honey samples with a higher abundance in honey harvested later in the year.

### 3.3. Urban and Suburban Sites

Pollen diversity in honey from Vienna was generally high, which is in accordance with other studies showing the effect of urbanization on the quantity and diversity of pollen in urban honey [30]. Although the flight radius of honeybees in urban and suburban apiaries comprised varying land covers (Figure 8), taxa richness in honey did not significantly differ between the two groups (167 vs. 164 identified taxa, respectively). Mean taxa richness and mean Shannon Diversity Index per sample did not significantly differ either (Figure 1A,B), but taxa composition did (Figure 1C). This was described before by [1] for species richness and composition identified by DNA-metabarcoding of honey.

On the other hand, in this study, the composition of foraged plants differed greatly between samples within the urban and suburban groups, respectively. This was reproducible over the years. Honeybee colonies preferred unique plant taxa even though their flight radius overlapped largely with the 3 km circle around other apiaries. The median estimated flight radius of honeybees in urban areas was shown to be even shorter than in non-urban areas [31,32]. The high heterogeneity of green patches found in cities leads to a more diverse array of foraging plants. Hence, potential food sources are available in even closer proximity to the hive. This might be especially true for mass-foraging plants like trees.

Taxa richness peaked in areas where the flight radius covers both urban and suburban areas and/or stretches beyond the city borders. In the 18th district, the apiary is situated on university grounds in a residential neighborhood next to a large park. The flight radius also covers vineyards and broadleaf forests. There, the number of identified taxa ranged from 44 to 65 over the three years. In the dense urban locations, such as in the 1st and the 15th districts, it was lower. With smaller parks, few private gardens, and fewer recreational areas compared to the suburbs, there are also fewer floral resources available for pollinators. The airport lies outside of the municipal borders but likewise consists of large concrete areas. Within the flight radius are large dry grasslands, agricultural fields, and the riparian forest of the Donau-Auen National Park.

Pollen spectra of specific locations in urban and suburban areas show that the dominance of certain taxa diminishes toward the outskirts. *Ailanthus altissima*, for example, was the most abundant pollen type in the 1st and 15th districts in all three years, with a mean of 53.1% and 32.8%, respectively. In the 18th district, its mean abundance was 12.2%, and at the airport, it was 11.6%. For honeybees, therefore, mass foraging resources might be described as a gradient from the predominance of *Ailanthus altissima* in the concrete inner city towards the predominance of *Brassica* sp. in suburban to rural areas.

### 3.4. Non-Native Plants May Benefit Foragers

Alien plants are an important food source for bees, especially when they dominate the “floral market” through high relative densities and abundances [17]. Together with the managed native vegetation, they may have a powerful effect on plant–pollinator interactions in urban habitats [3]. For the melissopalynological analysis of honey, however, alien plants might pose specific challenges. In addition to knowledge about the native flora of a region, the identification process requires access to data on what is actually planted in cities.

As the climate is changing and summers become warmer and dryer, municipal and private gardeners have to adapt their planting regimes. They may select plant species and plant compositions that are known to withstand the predicted conditions [19]. Oftentimes, this includes plant taxa that are closely related to native species. One example is *Fraxinus ornus* (Figure 5C,D), which has its natural distribution towards the South and the Eastern Mediterranean. In urban areas, it is slowly replacing the native *Fraxinus excelsior*. While the latter is strictly wind-pollinated, *Fraxinus ornus* depicts a double pollination strategy and is commonly foraged by honeybees, similar to *Castanea sativa* [33]. Further non-native *Fraxinus* species planted in Vienna include *F. angustifolia*, *F. americana*, and *F. pennsylvanica*. The latter is also considered to be an invasive neophyte [34].

Other plant genera have not had representatives in Europe in modern times. *Gleditsia triacanthos* and *Liriodendron tulipifera* are major sources of nectar for honeybees in the Eastern parts of North America and have become common urban trees in cities across Western and Central Europe. Similarly popular are *Koelreuteria paniculata*, *Sophora japonica*, and *Tetradium danielii*, which all originate in Eastern Asia. While the honey value of many native tree species has been described [18,35], information on nectar production and nutritional quality of many non-native ornamental trees has rarely been researched so far [36].

Extensive observations of plant–pollinator interactions have been conducted on urban and ornamental alien plants. However, they usually neglect trees, which, as this study shows, are the major foraging source in inner city environments. Ref. [37] concluded that bee abundance and insect species richness were higher in plots of non-native herbaceous plants. Native plots, on the other hand, would host more specialized pollinators. As [38] discovered, the majority of nectar-producing herbaceous plants and shrubs in urban areas were non-native. Some produced more nectar sugar than native plants, e.g., *Lavandula*, *Nepeta,* and *Mahonia*. Important nectar-producing shrubs were *Berberis*, *Buddleja*, and *Ceanothus* [38]. All of these plants commonly grow in Vienna’s green spaces. Single grains of *Berberis* sp. (Figure 7Q,R) and an unidentified 6-aperturate Lamiaceae were present in honey from Vienna, together with pollen from *Buddleja* sp. (Figure 7C,D), which occurred in 29 out of 50 samples (Table 2). Preliminary data from other Central European cities suggest that *Buddleja* might be a major foraging source for honeybees in urban areas nonetheless [39].

### 3.5. Multiple Factors Influence Taxa Richness

The amount of identified plant taxa varied over the years and did not correlate with the numbers of analyzed honey samples. In 2021, 141 taxa were identified from 20 honey samples at 16 apiary locations, whereas in 2022, 142 taxa were identified from only 11 samples in 11 locations (Table 1). This indicates that taxa richness in urban honey is determined by parameters other than solely location.

One of the most relevant parameters for the foraging behavior of pollinators is the annual weather conditions. Honeybees do not forage in very cold, rainy, or windy conditions [23]. Firstly, foraged flowers only open fully and display their pollen for bees in adequate weather. Secondly, bad conditions impact the honeybees’ ability to fly and navigate to the floral source and back to the colony [40]. In 2020 and 2021, the average number of taxa per sample was similar, but taxa composition differed. In the year 2020, the weather in spring was more favorable than in summer, allowing honeybees to forage more spring bloomers, e.g., *Salix* sp., *Acer* sp., and *Prunus* species. In the year 2021, weather conditions were especially unfavorable in springtime but suitable in summer, leading to higher numbers of taxa flowering in June and July. In 2022, there was a considerable increase in taxa richness per sample. Honeybees encountered suitable foraging conditions in spring as well as in summer, leading to the most complete pollen spectra in honey considering the phenological progression.

Another crucial parameter for taxa richness in honey from cities is specific annual planting regimes by private and municipal gardeners. This affects mostly annual and perennial herbaceous species, as well as recently planted trees and shrubs. Oftentimes, planting regimes follow certain recommendations, such as the ability to adapt to urban heat islands [19]. They might also follow the aesthetic sense of planners and landscapers. Similarly, the existence and frequency of unintentional weeds on brownfields and road verges fluctuate over the years. This is due to the high dynamic in planning and construction, in addition to the intensity of chemical and mechanical weed control [2,16,32,41].

Appropriately managed urban green spaces can act as hotspots for pollination services by bees [7]. However, the frequency of mowing and cutting hedges determines the floral availability in tended green spaces. For aesthetic reasons, lawns in inner-city environments are often cut at least once a week, inhibiting herbaceous mass foraging plants such as *Trifolium repens* from developing flower heads. In addition, shrubs such as *Ligustrum vulgare*, *Buxus sempervirens*, and *Ilex aquifolium* are regularly cut into shape, which results in the removal of inflorescences.

### 3.6. Outlook

Pollen composition in urban honey will likely further change with adaptations of planting regimes to more heat- and drought-resistant taxa. This will likely lead to even more non-native taxa and/or higher abundances of pollen from non-native taxa. Trees hereby have an important synergistic role in urban environments. They require relatively low long-term maintenance compared to herbaceous flower beds, they have a shading effect, and they provide ornamental value to inhabitants of cities. By choosing entomophilous mass-flowering taxa, gardeners can increase and provide foraging opportunities for honeybees throughout future years. For apiarists, the high amounts of non-native trees may benefit honeybee fitness and create new types of monofloral honey in the future. Further studies on this topic should consider the highly dynamic nature of urban beekeeping (e.g., shifts in apiary locations) as well as the specific weather conditions and, potentially, maintenance regimes over the study period.

## 4. Materials and Methods

### 4.1. Study Design

**Figure 10:**
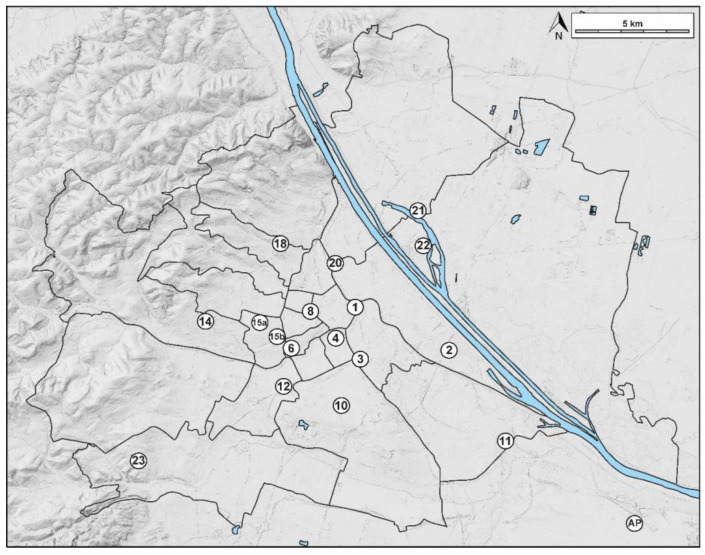
Overview of all 18 locations (numbers according to the respective administrative districts of Vienna, AP = Airport).

For this study, beekeepers were asked to send extracted honey samples from their apiaries within the city of Vienna for three consecutive years. The samples comprised a total of 17 locations in 16 out of 23 municipal districts. In addition, honey from one apiary at the Vienna International Airport was included, which is located approximately 2 km southeast of the municipality border and 20 km from the city center of Vienna. For a complete list of locations and the number of honey samples received per year, see Table 4 and the overview map (Figure 10).

Additionally, four apiary locations were chosen for a more detailed comparison of pollen diversities. These were locations 1 and 15a as representatives of densely built-up urban areas, location 18 as northwestern outskirts within the municipality line, and the airport.

### 4.2. Materials

All honey samples were extracted by apiarists and received in 240–500 g glass jars. Each sample was from an individual apiary with a minimum of three bee hives. At seven locations, a complete set of samples from three years (2020, 2021, and 2022) was extracted. At the other locations, honey could only be obtained from one or two of these years. This was due to bad weather conditions limiting honey production or because locations were shifted or abandoned by the apiarists. In the majority of apiaries, honey was harvested one time in the first half of July. At five locations, two harvests were possible (early June and late July to August). All of the delivered jars were either labeled as multifloral honey or they were not labeled at all.

### 4.3. Sample Preparation

All samples were prepared and analyzed according to the harmonized methods of melissopalynology [11,42]. They were thoroughly homogenized before approximately 5–6 g of honey material was dissolved in 10 mL of deionized water in a small beaker on a magnetic stirrer. When fully dissolved, the samples were transferred into a 12 mL pointed glass tube, centrifuged at 3000 rpm (1107× *g*) for three minutes, and decanted. This was repeated a second time to completely finish off the dissolved sample. After the second decanting, the glass tubes were left upside down on a drying rag for approximately 10 min. With a pipette, the sugary deposit on top of the pollen pellet was removed. After 100 µL of deionized water was added, the resuspended pollen pellet was pipetted onto an object slide and dried on a heating plate at 40 °C. Lastly, a drop of unstained glycerol jelly was applied, and the samples were covered with a 20 × 20 mm cover glass and left to rest for at least 24 h.

### 4.4. Melissopalynological Analysis

Both identification and counting were conducted at 1000-x magnification in oil, using an Olympus BX50 light microscope with an attached digital camera. If necessary, online pollen databases *PalDat* and Pollen-Wiki, as well as other relevant literature, were consulted [43,44,45,46]. To examine the relative abundances of the contained pollen types, individual grains were counted out to a total amount of 500 per sample. While pollen of anemophilous species such as Poaceae and Pinaceae were included (as advised by [47]), spores and algae were not. The relative abundances were later assigned to the following categories: predominant (>45%), secondary (15–45%), important minor (3–15%), and minor pollen types (<3%). In addition, a threshold of 8% was set to determine the overall most abundant taxa in this study.

### 4.5. Ecology of Foraging Plants

For information on the growth type of most taxa, [48] was consulted. The botanical status of taxa in Austria was retrieved from the List of Neobiota in Austria [34] and integrated into four new individual categories relevant to present and future foraging plants: native/established, invasive, ornamental, and agricultural. Refs. [36,49] provided information on the nectar value of common native and ornamental plant species.

### 4.6. Land Cover Analysis

QGIS v.3.16.4-Hannover [50] was used to produce an overview map of the 18 locations. The percentual land cover in a 3 km flight radius was calculated based on the “CORINE Land Cover 2018 (raster 100 m), Europe, 6-yearly” dataset [51].

### 4.7. Statistical Exploitation

Data preparation and analysis were performed with R [52], and graphics were created using the ggplot2 package [53]. The calculation of the Shannon Diversity Index, permutational multivariate analysis of variance, and principal component analysis for taxa composition were carried out using the functions of the vegan package [54].

## 5. Conclusions

Urban green spaces can provide synergistic benefits for pollinators, apiarists, and city dwellers alike. As a source of nectar and pollen, trees provide mass foraging opportunities for pollinators. This study suggests that the disproportionally high relative abundance of tree pollen from a mixture of European, East Asian, and North American tree species is typical for honey from a Central European city. The most predominant pollen type was *Ailanthus altissima*, which occurred in decreasing abundances from the densely built-up urban center towards the outskirts. General taxa richness did not differ between urban and suburban areas. From a palynological point of view, the increasing popularity of urban beekeeping brings up new challenges for the analysis of urban honey in terms of botanical and geographical origin. The results of this study contribute to the understanding of honeybee foraging patterns in cities and might help decision-makers improve the management of green spaces for pollinators.

## Figures and Tables

**Table 1:** Number of samples, apiary locations, and identified taxa per year.

Year	*n* Samples	*n* Locations	*n* Taxa
2020	19	15	150
2021	20	16	141
2022	11	11	142

**Table 2:** The 33 pollen types occurring in more than 50% of all analyzed samples, their growth habit (T = tree, S = shrub, H = herbaceous, L = liana, E = epiphyte), number of samples, number of samples per frequency category (predominant = > 45%, secondary = 15–45%, important minor = 3–15%, minor = < 3%), and maximum relative abundance in %.

Taxa Name	Family	Growth Type	*n* Samples	> 45%	15–45%	3–15%	<3%	Max. (%)
*Ailanthus altissima*	Simaroubaceae	T	49	15	20	10	4	80.60
*Tilia* sp.	Malvaceae	T	49	0	5	30	14	37.20
*Aesculus hippocastanum*	Sapindaceae	T	48	0	2	18	28	30
*Castanea sativa*	Fagaceae	T	47	1	2	22	22	49
*Gleditsia triacanthos*	Fabaceae	T	47	0	1	23	23	39.60
*Parthenocissus* sp.	Vitaceae	L	47	0	0	22	25	11.20
*Syringa* sp.	Oleaceae	S	47	0	0	12	35	8.20
*Acer* sp.	Sapindaceae	T	46	0	3	19	24	19.20
*Plantago* sp.	Plantaginaceae	H	46	0	0	1	45	3.60
*Trifolium repens*-Type	Fabaceae	H	46	0	0	4	42	6.80
*Sambucus*-Type	NA	S	46	0	0	9	37	5.20
Malus-Type	Rosaceae	T	44	0	0	2	42	11.80
unidentified	unidentified	NA	43	0	0	0	43	2.20
*Anemone*-Type	Ranunculaceae	H	39	0	0	1	38	3.20
*Koelreuteria paniculata*	Sapindaceae	T	38	0	0	5	33	4.20
Poaceae	Poaceae	H	37	0	0	1	36	3
Brassicaceae	Brassicaceae	H	36	0	0	7	29	13.80
*Cornus sanguinea*	Cornaceae	S	36	0	0	1	35	5.60
*Salix* sp.	Salicaceae	T	34	0	0	3	31	9.60
*Rubus* sp.	Rosaceae	S	34	0	0	2	32	9.60
*Prunus* sp.	Rosaceae	T	33	0	1	3	29	31.80
*Myosotis* sp.	Boraginaceae	H	32	2	1	12	17	64.80
*Fragaria* sp./*Potentilla* sp.	Rosaceae	H	32	0	0	1	31	4
*Echium* sp.	Boraginaceae	H	31	0	0	6	25	9.20
*Allium* sp.	Alliaceae	H	30	0	0	3	27	10.80
*Buddleja* (4-aperturate)	Scrophulariaceae	S	29	0	0	3	26	6.40
*Sophora japonica*	Fabaceae	T	28	2	1	6	19	63
*Taraxacum*-Type	Asteraceae	H	28	0	0	0	28	1
*Begonia* sp.	Begoniaceae	H	28	0	0	1	27	3
*Robinia pseudoacacia*	Fabaceae	T	27	0	0	0	27	1.60
Rosaceae	Rosaceae	NA	26	0	0	0	26	2
Lamiaceae (6-aperturate)	Lamiaceae	H	25	0	0	0	25	0.60
*Liriodendron tulipifera*	Magnoliaceae	T	25	0	0	0	25	1

**Table 3:** Pollen of non-native ornamental tree species (19) and their biogeographical origin, as well as the number of samples in which they occurred, the maximum relative abundance (max. %), and whether the plants produce nectar.

Taxa Name	Family	Origin	*n* Samples	Max. (%)	Nectar
*Catalpa* sp.	Bignoniaceae	North America, East Asia	8	0.4	yes
*Celtis* sp.	Cannabaceae	Southern Europe, North America	1	0.2	no
*Corylus colurna*	Betulaceae	South-Eastern Europe, Asia	6	0.6	no
*Davidia involucrata*	Nyssaceae	East Asia	1	1	yes
*Fraxinus ornus*	Oleaceae	Mediterranean	20	1.8	yes
*Ginkgo biloba*	Ginkgoaceae	East Asia	4	0.4	no
*Gleditia triacanthos*	Fabaceae	North America	47	39.6	yes
*Koelreuteria paniculata*	Sapindaceae	East Asia	38	4.2	yes
*Liquidambar styraciflua*	Altingiaceae	North America	1	0.2	yes
*Liriodendron tulipifera*	Magnoliaceae	North America	25	1	yes
*Morus* sp.	Moraceae	South-Eastern Europe	11	2	no
*Paulownia tomentosa*	Paulowniaceae	East Asia	2	1	yes
*Platanus* sp.	Platanaceae	Southern Europe, Asia	20	1	no
*Pterocarya fraxinifolia*	Juglandaceae	Caucasus	2	0.2	no
*Ptelea trifoliata*	Rutaceae	North America	1	0.2	yes
*Sophora japonica*	Fabaceae	East Asia	28	63	yes
*Tamarix* sp.	Tamaricaceae	Southern Europe, Asia, Africa	3	5.6	yes
*Tetradium danielii*	Rutaceae	East Asia	12	1.2	yes
*Zelkova serrata*	Ulmaceae	East Asia	1	0.4	no

**Table 4:** List of apiaries with the number of analyzed samples per year and their surroundings classified as urban and suburban, respectively.

Sample	District	Location	2020	2021	2022	Proximity
25	1st	Stubenring	1	1	1	Urban
31	3rd	Hotel Daniel	2	2	-	Urban
44	4th	Technical University of Vienna	-	1	1	Urban
39	6th	ibis Hotel	1	1	-	Urban
26	8th	Palais Auersperg	1	1	1	Urban
36	10th	Suchenwirtplatz	1	1	1	Urban
27	12th	Eichenstraße	1	1	1	Urban
28	15th a	Boutiquehotel Stadthalle	1	1	1	Urban
32	15th b	KGV Schmelz	2	2	-	Urban
46	20th	Donaukanal	-	1	1	Urban
33	2nd	Prater Lusthaus	2	2	-	Suburban
45	11th	Gärtnerei Auer	-	1	1	Suburban
40	14th	Flötzersteig	1	2	-	Suburban
29	18th	University of Natural Resources and Life Sciences	1	1	1	Suburban
59	21st	An der Schanze	1	-	-	Suburban
30	22nd	NH Danube City Hotel	1	-	1	Suburban
34	23rd	Pappelteich	2	1	-	Suburban
37	AP	Vienna International Airport	1	1	1	Suburban

## Data Availability

All data supporting the findings of this study are either part of the published manuscript or available from K.K. upon reasonable request.

## References

[1] Lucek K., Galli A., Gurten S., Hohmann N., Maccagni A., Patsiou T., Willi Y. (2019). Metabarcoding of honey to assess differences in plant-pollinator interactions between urban and non-urban sites. Apidologie.

[2] Baldock K.C.R., Goddard M.A., Hicks D.M., Kunin W.E., Mitschunas N., Morse H., Osgathorpe L.M., Potts S.G., Robertson K.M., Scott A.V. (2019). A systems approach reveals urban pollinator hotspots and conservation opportunities. Nat. Ecol. Evol..

[3] Harrison T., Winfree R. (2015). Urban drivers of plant-pollinator interactions. Funct. Ecol..

[4] Potts S.G., Biesmeijer J.C., Kremen C., Neumann P., Schweiger O., Kunin W.E. (2010). Global pollinator declines: Trends, impacts and drivers. Trends Ecol. Evol..

[5] Goulson D., Nicholls E., Botías C., Rotheray E.L. (2015). Bee declines driven by combined stress from parasites, pesticides, and lack of flowers. Science.

[6] Hall D.M., Camilo G.R., Tonietto R.K., Ollerton J., Ahrné K., Arduser M., Ascher J.S., Baldock K.C.R., Fowler R., Frankie G. (2017). The city as a refuge for insect pollinators. Conserv. Biol..

[7] Theodorou P., Radzevičiūtė R., Lentendu G., Kahnt B., Husemann M., Bleidorn C., Settele J., Schweiger O., Grosse I., Wubet T. (2020). Urban areas as hotspots for bees and pollination but not a panacea for all insects. Nat. Commun..

[8] Moosbeckhofer R. Autochthone Bienenrassen in Österreich. Proceedings of the Biodiversität in Österreich Conference, Höhere Bundeslehr-und Forschungsanstalt für Landwirtschaft.

[9] Crane E. (1990). Bees and Beekeeping: Science, Practice and World Resources.

[10] Johnson B.R. (2023). Honey Bee Biology.

[11] von der Ohe W., Persano Oddo L., Piana M.L., Morlot M., Martin P. (2004). Harmonized methods of melissopalynology. Apidologie.

[12] Halbritter H., Ulrich S., Grimsson F., Weber M., Zetter R., Hesse M., Buchner R., Svojtka M., Frosch-Radivo A. (2018). Illustrated Pollen Terminology.

[13] Cho Y., Lee D. (2018). ‘Love honey, hate honey bees’: Reviving biophilia of elementary school students through environmental education program. Environ. Educ. Res..

[14] Noël G., Mestrez A., Lejeune P., Francis F., Kawai J., Miwa M., Uehara K., Nagase A. (2023). Pollen meta-barcoding reveals different community structures of foraged plants by honeybees (*Apis mellifera* L.) along space-time gradient in Japan. Urban For. Urban Gree..

[15] Sponsler D.B., Shump D., Richardson R.T., Grozinger C.M. (2020). Characterizing the floral resources of a North American metropolis using a honey bee foraging assay. Ecosphere.

[16] Lepczyk C., Aronson M., Evans K., Goddard M., Lerman S., MacIvor J.S. (2017). Biodiversity in the City: Fundamental Questions for Understanding the Ecology of Urban Green Spaces for Biodiversity Conservation. BioScience.

[17] Williams N.M., Cariveau D., Winfree R., Kremen C. (2011). Bees in disturbed habitats use, but do not prefer, alien plants. Basic Appl. Ecol..

[18] Somme L., Moquet L., Quinet M., Jaquemart A.L. (2016). Food in a row: Urban trees offer valuable floral resources to pollinating insects. Urban Ecosyst..

[19] Sjöman H., Gunnarsson A., Pauleit S., Bothmer R. (2012). Selection Approach of Urban Trees for Inner-city Environments: Learning from Nature. Arboric. Urban For..

[20] Little E.L. (1979). Checklist of United States Trees (Native and Naturalized).

[21] Magistrat der Stadt Wien (2022). Statistisches Jahrbuch der Stadt Wien. *MA23 Wirtschaft, Arbeit und Statistik*. https://www.digital.wienbibliothek.at/wbrup/periodical/pageview/4357365.

[22] Magistrat der Stadt Wien (2024). Wien Umweltgut: Baumkataster. *MA42 Wiener Stadtgärten*. https://data.wien.gv.at.

[23] Moosbeckhofer R., Bretschko J. (1996). Naturgemäße Bienenzucht.

[24] Brodschneider R., Gratzer K., Kalcher-Sommersguter E., Heigl H., Auer W., Moosbeckhofer R., Crailsheim K. (2019). A citizen science supported study on seasonal diversity and monoflorality of pollen collected by honey bees in Austria. Sci. Rep..

[25] Ropars L., Dajoz I., Fontaine C., Muratet A., Geslin B. (2019). Wild pollinator activity negatively related to honey bee colony densities in urban context. PLoS ONE.

[26] Thompson J.S. (2008). Pollination Biology of *Ailanthus altissima* (Mill.) Swingle (Tree-of-Heaven) in the Mid-Atlantic United States. Ph.D. Thesis.

[27] Poljuha D., Uzelac M., Zubin Ferri T., Damijanić D., Šimunić M., Korovljević H., Weber T., Sladonja B. (2008). Morphology of extrafloral nectaries of *Ailanthus altissima* (Mill.) Swingle (Simaroubaceae). Period. Biol..

[28] Persano Oddo L., Piana L., Bogdanov S., Bentabol A., Gotsiou P., Kerkvliet J., Martin P., Morlot M., Ortiz Valbuena A., Ruoff K. (2004). Botanical Species giving unifloral honey in Europe. Apidologie.

[29] Gardi T., Micheli M., Petrarchini M. (2020). *Ailanthus Altissima* (Mill.) and Varroa Destructor (Anderson & Trueman)—Two Alien and Invasive Species with Impact on the Environment and on the “Hive System”. World J. Agri. Soil Sci..

[30] Gamrat R., Puc M., Gałczyńska M., Bosiacki M., Witczak A., Telesiński A. (2022). Differences in the Pollen Content of Varieties of Polish Honey from Urban and Rural Apiaries. Acta Univ. Cibiniensis Ser. E Food Technol..

[31] de Vere N., Jones L.E., Gilmore T., Moscrop J., Lowe A., Smith D., Hegarty M.J., Creer S., Ford C.R. (2017). Using DNA metabarcoding to investigate honey bee foraging reveals limited flower use despite high floral availability. Sci. Rep..

[32] Fox G., Vellaniparambil L.R., Ros L., Sammy J., Preziosi R.F., Rowntree J.K. (2022). Complex urban environments provide *Apis mellifera* with a richer plant forage than suburban and more rural landscapes. Ecol. Evol..

[33] Giovanetti M., Aronne G. (2011). Honey bee interest in flowers with anemophilous characteristics: First notes on handling time and routine on *Fraxinus ornus* and *Castanea sativa*. Bull. Insectology.

[34] Essl F., Rabitsch W. (2002). Neobiota in Österreich.

[35] Milecka M., Kot N., Malawski S. (2019). Historical gardens and their potential use for proecological development of urban green spaces, as illustrated by Felin Manor Park in Lublin, Poland. Acta Agrobot..

[36] Radtke J., Schirm P. (2015). Bienenweide.

[37] Seitz N., van Engelsdorp D., Leonhardt S.D. (2020). Are native and non-native pollinator friendly plants equally valuable for native wild bee communities?. Ecol. Evol..

[38] Tew N.E., Memmott J., Vaughan I.P., Bird S., Stone G.N., Potts S.G., Baldock K.C. (2021). Quantifying nectar production by flowering plants in urban and rural landscapes. J. Ecol..

[39] Koelzer K. Pollen Analyses of Urban Honey from Various Cities in Central Europe, 2020–2022.

[40] Goodman L. (2022). Form and Function in the Honey Bee.

[41] Lowenstein D.M., Matteson K.C., Minor E.S. (2019). Evaluating the dependence of urban pollinators on ornamental, non-native, and ‘weedy’ floral resources. Urban Ecosyst..

[42] Koelzer K., Ribarits A., Weber M. (2024). Comparing the acetolysed and hydrated methods for the pollen analysis of honey. Grana.

[43] PalDat—A Palynological Database. www.paldat.org.

[44] Stebler T. Pollenatlas: Pollen-Wiki. https://pollen.tstebler.ch/MediaWiki/index.php?title=Pollenatlas.

[45] Beug H.J. (2004). Leitfaden der Pollenbestimmung für Mitteleuropa und Angrenzende Gebiete.

[46] Reille M. (1992). Pollen et Spores D’europe et D’afrique du Nord.

[47] Saunders M.E. (2018). Insect pollinators collect pollen from wind-pollinated plants: Implications for pollination ecology and sustainable agriculture. Insect Conserv. Diver..

[48] Martensen H.O., Probst W. (1990). Farn- und Samenpflanzen in Europa.

[49] Pritsch G. (2007). Bienenweide.

[50] QGIS Development Team QGIS Geographic Information System. Open Source Geospatial Foundation Project. http://qgis.osgeo.org.

[51] Copernicus Land Monitoring Service (2020). CORINE Land Cover 2018 (Raster 100 m), Europe, 6-yearly, Dataset. DOI (Raster 100 m).

[52] R Core Team R: A Language and Environment for Statistical Computing. R Foundation for Statistical Computing. https://www.R-project.org.

[53] Wickham H. (2016). ggplot2: Elegant Graphics for Data Analysis.

[54] Oksanen J., Blanchet F.G., Kindt R., Legendre P., O’hara R.B., Simpson G.L., Solymos P., Stevens M.H., Wagner H. vegan: Community Ecology Package. R Package Version 2.6-4, 2022. https://CRAN.R-project.org/package=vegan.

